# Biological volume EM with focused Ga ion beam depends on formation of radiation-resistant Ga-rich layer at block face

**DOI:** 10.21203/rs.3.rs-5235218/v1

**Published:** 2025-07-10

**Authors:** Richard Leapman, Zijie Yang, Joshua Kim, Guofeng Zhang, Maria Aronova

**Affiliations:** National Institutes of Health; National Institutes of Health; National Institutes of Health; National Institute of Biomedical Imaging and Bioengineering; National Institutes of Health

## Abstract

Volume electron microscopy (vEM) enables biologists to visualize nanoscale 3D ultrastructure of entire eukaryotic cells and tissues prepared by heavy atom staining and plastic embedding. The highest resolution vEM technique is focused ion-beam scanning electron microscopy (FIB-SEM), which provides nearly isotropic (~5–10 nm) spatial resolution at fluences of > 10,000 e^−^/nm^2^. However, it is not clear how such high resolution is achievable because serial block-face (SBF) SEM, which incorporates an in-situ ultramicrotome instead of a Ga^+^ FIB beam, results in radiation-induced collapse of similar specimen blocks at fluences of only ~20 e^−^/nm^2^. Moreover, the z-resolution (perpendicular to block-face) in FIB-SEM is substantially better than predicted by Monte Carlo modeling of SBF-SEM samples. Here, we show that FIB-SEM implants a thin highly concentrated layer of Ga^+^ ions at the block surface, which greatly reduces the effect of beam-damage, reduces the depth from which backscattered electrons are detected, and prevents specimen charging and collapse.

## Introduction

The advent of techniques for 3D volume electron microscopy (vEM) over the past two decades has transformed ultrastructural imaging of eukaryotic cells and tissues^1–4^. These methods require that the specimen be fixed, stained with heavy elements, and embedded in polymerized plastic. Ultrastructure can then be imaged in 3D from volumes ranging from 10^4^ μm^3^ (i.e., single eukaryotic cells) to 10^9^ μm^3^ of tissues. The journal *Nature* featured vEM as one of its seven technologies to watch in 2023, as tools and techniques that are poised to have an outsized impact on science in the coming year^5^. The highest resolution of these techniques suitable for imaging entire eukaryotic cells and tissues at a near isotropic resolution of ~10 nm is focused ion beam scanning electron microscopy (FIB-SEM), in which a Ga^+^ ion beam ablates very thin (3–10 nm) layers from a specimen block face, while a stack of backscattered images is collected^6,7^. Such data sets reveal heavy atom-stained ultrastructure in 3D at a resolution of 5–10 nm. However, the reason why FIB-SEM is indeed capable of providing such high-resolution visualization of cellular structure has not yet been fully explained. Specifically, several aspects of the data collection in the FIB-SEM have appeared inconsistent with what is known about the interactions between the electron probe and plastic-embedded stained biological specimens. Specifically, why can the specimen blocks withstand such high electron fluences^8^ (>10^4^ e/nm^2^) without physical collapse? Why is the structural information contained within the surface layer^9^ of 5–10 nm for 1.5 keV primary electrons? And why doesn’t surface charge build-up^10^ occur? Here we have performed both experiments and simulations of electron scattering that resolve these inconsistencies by the discovery of highly concentrated Ga^+^ implantation within a 25 nm surface layer of the block.

To understand how this phenomenon occurs, it is helpful to consider briefly the various vEM techniques that are now beginning to find wide use. In the automated tape-collecting ultramicrotome (ATUM) thin sections are cut in the form of ribbons that are transferred first to tape and then to silicon wafers^4^; these arrays of sections are imaged in the scanning electron microscope (SEM) using a backscattered electron detector that is sensitive to local concentrations of heavy-atom stain (Os, Pb, and U), which provides contrast from the 3D cellular ultrastructure, equivalent to that obtained by conventional transmission electron microscopy (TEM). Although the ATUM enables the acquisition of very large ultrastructural volumes, e.g., cubic millimeters of brain tissue, with a lateral (x, y) spatial resolution of ~5 nm, the spatial resolution in the z-direction (perpendicular to the block-face) is limited to ~30 nm, due to the minimum slice thickness that can be cut by the diamond knife of an ultramicrotome.^4^

An alternative to the ATUM is serial block-face SEM (SBF-SEM)^11–16^, in which an ultramicrotome is incorporated into the specimen stage of the SEM, enabling thin slices to be removed successively from the block-face *in situ*, in the vacuum of the SEM. In SBF-SEM the thin sectioned slices are discarded, and the fresh block-face is imaged using the backscattered signal generated by the electron beam whose energy is typically between 1.2 and 1.5 keV. The SBF-SEM has the advantage of avoiding distortions of the sections that can occur in the ATUM, but the z-resolution is again limited by the minimum slice thickness that can be removed from the block (i.e., ~30 nm). It is also found that the block-face in the SBF-SEM is prone to electrical charging that distorts the images, although this can now be mitigated by use of a focused charge compensation device that neutralizes the charge build-up. A bigger limitation of the SBF-SEM is that the maximum electron fluence is only ~20 electrons per nm^2^, beyond which the surface of the block undergoes radiation damage resulting in collapse. This limits the signal-to-noise ratio for detecting ultrastructural features in the image, even though the contrast of those features is high. And importantly, Monte Carlo simulations show that 1.5 keV electrons generate a backscattered signal originating from a depth of at least 25 nm into the block, which predicts that the z-resolution of the FIB-SEM should only be ~25 nm if the Ga^+^ ion beam simply removed thinner slices of material from the block than the built-in diamond knife of the SBF-SEM. Thus, we expect the z-resolution of FIB-SEM to be limited even though some mitigation is possible by energy-filtering the backscattered signal to exclude electrons that originate from deeper in the specimen. This is achievable by using an in-lens energy selected backscattered (ESB) electron detector with a bias voltage applied to a grid situated in front of the detector to repel lower energy electrons that have lost energy by scattering deeper into the specimen block^17^, or by applying a bias voltage to the specimen itself so that electrons, which undergo energy loss, are excluded from entering the backscattered electron detector^18^.

Acquiring 3D ultrastructural images from plastic-embedded stained biological structures using FIB-SEM requires several *in-situ* preparation steps that are performed under vacuum in the specimen stage. To understand the interaction of the Ga^+^ and e^−^ beams with the specimen, it is useful to summarize the microscope set-up. First, it is necessary to create a block face in the plane of the focused ion beam, and to ensure that this block-face is accessible to the scanning electron beam. In the Zeiss Crossbeam 550 FIB-SEM used in this study, the FIB beam is at an angle of 54° to the electron beam. The specimen block’s surface is therefore tilted to an angle of 54° towards the FIB beam, and a block-face perpendicular to the block’s surface is prepared for imaging. This requires deposition of a protective pad of a heavy element (platinum in our instrument) using the FIB beam to decompose an evaporated organometallic Pt compound. The protective pad avoids non-uniform ion beam milling, which is also referred to as ‘curtaining’^19^. The pad also allows the fabrication of registration marks by removing thin diagonally oriented grooves in the plane of the deposited Pt, which are then filled with carbon from an organic liquid released into the vacuum of the FIB-SEM to provide a slice-by-slice calibration marker that enables direct measurement of distance perpendicular to the block-face during the FIB-SEM volume acquisition.

**Figure 1:**
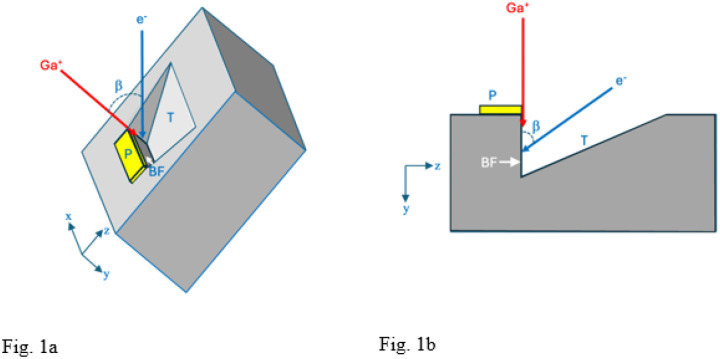
Principle of FIB-SEM, as applied to fixed, stained, and embedded biological specimen block. **(a,b)** Schematic illustrations showing, respectively, the *in-situ* preparation geometry in three dimensions (lateral x, height y, and depth z), and in two dimensions (as viewed along the x-axis in the y-z plane). The specimen block is tilted to angle b, enabling an incident 30-keV Ga^+^ beam at angle b from the vertical axis of the electron column to mill a trench (T), which allows geometrical access to the incident 1.5-keV electron probe (e^−^) as well as collection of backscattered electrons by an energy-selected backscattered (ESB) detector (**a**). A volume of the block is selected, on top of which a micrometer-thick platinum pad(P) is deposited by scanning the focused ion beam while organometallic Pt is released into the vacuum of the SEM in proximity to the specimen block. Diagonal fiducial lines are then etched in the Pt pad by the ion beam and filled in with a layer of deposited carbon, which provides an internal calibration of the milling depth along the z-axis (not shown). The Pt pad avoids irregular cutting of the block-face (BF) by the ion beam. Although the trench is milled normal to the surface of the block, material released in the direction opposite to the uncut block forms a downward sloping surface ending at the foot of the block face (**b**). The milled trench is made narrower at the block face than at the start of the trench furthest from the block face, to facilitate collection of the backscattered electrons (BSEs). After the Pt pad is deposited and the trench has been prepared, the 3-D volume data is acquired by continuous, simultaneous operation of the crossed Ga^+^ and e^−^ beams. This geometry detailed in Narayan and Subramaniam^7^ is relevant to understating novel aspects of the Ga-implantation described in the present work.

Before the block-face can be exposed a trench has to be milled in the specimen block allowing access to the electron beam as illustrated in Fig. 1. The trench material is removed rapidly by increasing the FIB current to ~30 nA, which forms a triangular trench in the y-z plane caused by ejection of the plastic-embedding material along with Ga^+^ ions. After the trench is formed, the block-face is polished by reducing the ion current to ~700 pA, and thin layers are successively ablated from the region of interest.

**Figure 2:**
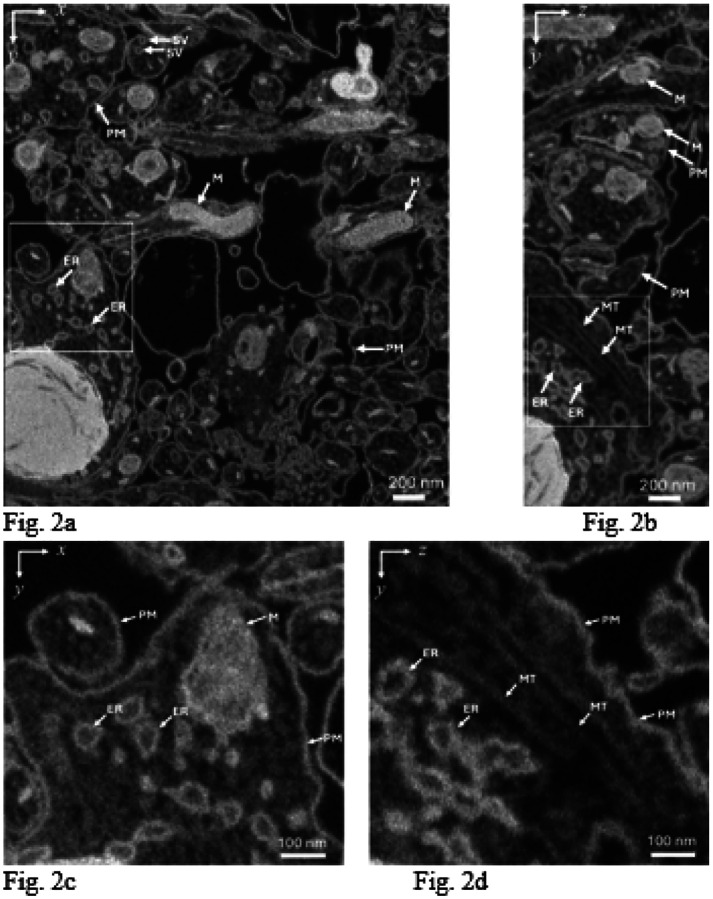
Slices through 3D data set acquired with the FIB-SEM from sample of cultured dissociated rat hippocampal neurons that were and fixed and stained before embedding in Araldite/Epon. **(a,b)** Representative orthoslices through the 3-D volume in the x-y plane (**a**) and in the y-z plane (**b**); PM, plasma membrane; SV, synaptic vesicle; M, mitochondrion; ER, endoplasmic reticulum; MT, microtubule. Higher magnification views of the ultrastructure in x-y plane (**c**) and y-z plane (**d**) indicate near-isotropic resolution of 3D FIB-SEM imaging data. Incident electron probe energy was 1.5 keV, probe current was 700 pA; grid voltage in front of the BSE detector was 700 V; pixel size was 3nm (in x, y, and z); pixel acquisition time was 16 μs for e^−^ beam; FIB Ga^+^ current was 700 pA, with concurrent scanning of the Ga^+^ and e^−^ beams. Examining the widths of membranes in the x-y plane (Fig. 2a) indicates an x-y resolution of approximately 5–10 nm, and in the y-z plane, the resolution in the z-direction is ~10 nm. In contrast, 3D data acquired using other vEM techniques, such as SBF-SEM and ATUM, typically show substantially degraded z-resolution relative to the x,y-resolution due to limitations of mechanical sectioning of the block (≳30 nm). However, as explained below, the observation of near-isotropic resolution in FIB-SEM is not predicted by Monte Carlo simulations of backscattered electron trajectories, and it can only be explained in terms of the Ga^+^ implantation.

We illustrate the 3D ultrastructure that is obtainable using FIB-SEM by data from a sample of cultured dissociated rat hippocampal neurons that were and fixed and stained before embedding in Araldite/Epon (Fig. 2). Examination of neurite plasma membranes, endoplasmic reticulum membranes, and synaptic vesicle membranes indicates widths of 5–10 nm in the x-y plane, and approximately 10 nm in the y-z plane, demonstrating a near-isotropic spatial resolution of ~10 nm throughout the volume, with a slightly degraded resolution in the z-direction, which is attributed to the penetration of the 1.5 keV electron probe ~10 nm into the block.

We expect that some Ga^+^ ions will be deposited in the layer close to the block-face, but it is not clear how many Ga^+^ ions are deposited in relation to the number of atoms contained in the Epon-Araldite polymer embedding the biological structures together with their bound stain. In the present study, we show that the excellent performance of the FIB-SEM for 3D imaging of cells and tissues is due to an important but serendipitous feature of the focused Ga^+^ beam acting on epoxy-resin embedded specimen blocks of stained biological specimens. We have examined the FIB-SEM block-face using: (1) the backscattered electron signal to view a cross section of the block in the SBF-SEM; (2) high-angle annular dark-field (HAADF) images from sections cut perpendicular to the block-face using scanning transmission electron microscopy (STEM); (3) electron energy loss spectroscopy (EELS) to obtain compositional information from the region of Ga^+^ implantation; and (4) we have compared experimental data with Monte Carlo simulations to model the backscattered electron signal from stained structures within the epoxy-embedded blocks.

## Results

### 1. Backscattered SEM electron images perpendicular to the block-face

**Figure 3:**
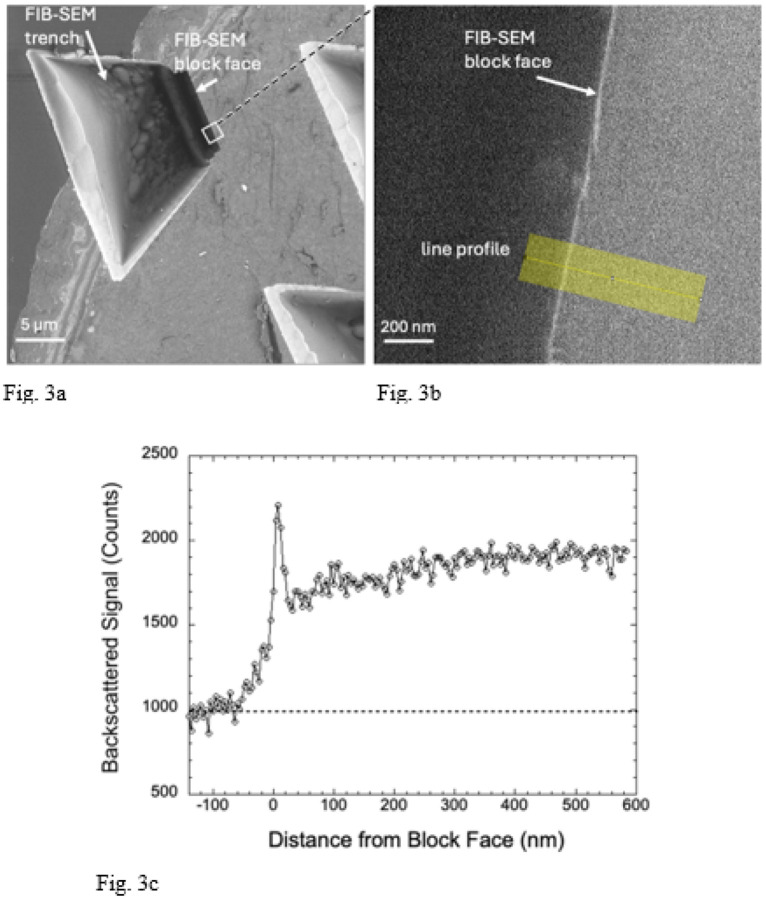
BSE images and line profile from FIB-SEM specimen block that had been transferred into a SBF-SEM, (a,b,c). Top view of trench at low magnification after removal of Pt pad by the *in-situ* diamond knife, showing location of block face at shorter end of trench. Portions of two other trenches are also present in the field (**a**). A higher magnification view of the block face contained in the white square in (**a**) showing a bright line approximately 20 nm in width at the edge, and position of line profile (**b**). Some regions of the block face appear to be fractured. The line profile of the BSE signal averaged along a 200-nm width perpendicular to the block face shows a sharp increase in signal at the edge with a signal-to-background ratio of about 1:1 (**c**).

We transferred specimen blocks that had been imaged in the FIB-SEM to an SBF-SEM, which enabled us to remove sections perpendicular to the FIB-SEM block face using the built-in ultramicrotome of the SBF-SEM. We first removed the protective Pt pad as well as at least 1 μm of the Epon-Araldite block beneath the pad to expose a freshly cut cross section through the FIB-SEM block face. At low magnification several trenches are visible (Fig. 3a). Backscattered electron images from these cross sections at higher magnification reveals a narrow line of width ~25 nm at the edge of the block (Fig. 3b), indicating a composition with higher atomic number than the surrounding Epon-Araldite embedding resin. The material at the block face boundary sometimes appeared to be fractured suggesting a hardened layer. A line profile from the region indicated by the yellow band in Fig. 3b and averaged over a width of 200 nm shows a sharp peak in the BSE signal at the FIB-SEM block face, which provides our first evidence that there is a well-defined layer of Ga^+^ ion implantation.

### 2. Analysis of HAADF-STEM images from thin sections cut perpendicular to FIB-SEM block face

**Figure 4:**
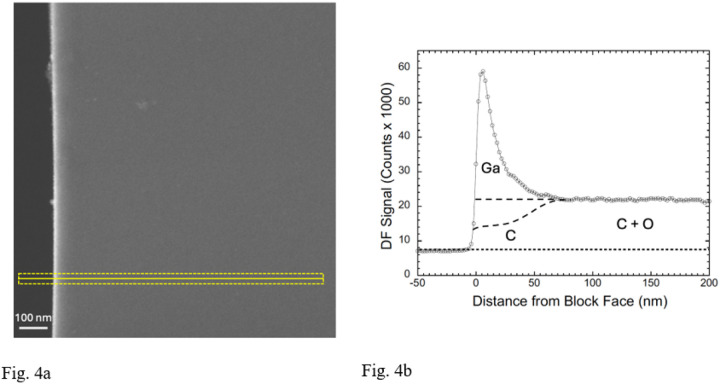
STEM-HAADF image of Epon section cut to thickness of ~200-nm with an ultramicrotome across a FIB-milled block face, and line profile in perpendicular direction. **(a,b)** HAADF image shows an bright intensity peak at the block face, across which an intensity plot was obtained perpendicular to the edge in the region shown (**a**). The line profile averaged over a 40-nm width parallel to the block face shows an asymmetrical, sharp peak of width ~20 nm with a sharp onset directly at the vacuum-block interface and a decay over distance of ~50 nm; the DF intensity then drops to a plateau corresponding to the signal from undamaged Epon resin (**b**). The profile retains a constant shape but moves to the right as the sample is ion milled. The higher intensity peak at the block face is attributed to Ga ions implanted in the block, and the plateau intensity far away from the block face is attributed to light atoms in the Epon, mainly C and O, since the elastic scattering cross section for H is so low. It is more difficult to ascribe the precise fraction of the DF signal that originates from C atoms (Supplementary Fig. 1, and Supplementary Note 1). The upper dashed line under the Ga peak in (**b**) corresponds to no C or O being lost from the region of Ga implantation, and the lower dashed line corresponds to 50% loss of C atoms and a complete loss of O atoms in the implantation region.

To explore the extent of Ga^+^ ion implantation quantitatively, we used an ultramicrotome to cut sections, of nominal thickness ~200 nm, through the FIB-SEM block faces in the regions of the Ga^+^ ion milled trenches. The sections were deposited on EM grids and transferred to a TEM-STEM operating at a beam energy of 300 keV. High-angle annular dark field (HAADF) images (Fig. 4a) were acquired with a pixel size of 2 nm. A higher contrast of the HAADF signal is immediately evident at the edge of the sectioned FIB-SEM block face, which is shown in the line profile (Fig. 4b) from the 40-nm wide region indicated in the lower part of Fig. 4a. The line profile provides quantitative information about the concentration of implanted Ga ions in the vicinity of the Epon-Araldite block face. The peak in the HAADF signal is very abrupt at the block face with a width of ~6 nm, even though the nominal thickness of the section is 30 times greater. The peak, which is attributed to gallium implantation, has an asymmetrical shape. We also observe an apparent decay over a length of ~60 nm, at which point the elevated HAADF signal drops off to the level observed throughout the interior of the specimen block, i.e., far away from the FIB beam. This decay of the peak is suggestive of diffusion of Ga atoms into the block. The peak signal in the Ga layer has a jump ratio relative to the signal in the interior of the block of ℛ~3.9:1. The HAADF detector is not sensitive to H atoms, so we cannot comment on the possible loss of H from the specimen. In addition, we do not know what fraction of C and O atoms have been lost from the region of the strong Ga peak at the FIB-SEM block face, but it is clear from Fig. 4b that most of the observed signal is due to scattering from Ga atoms.

It is possible that oxygen atoms are lost by interaction of the sectioned block face with the electron beam in the TEM-STEM rather than by interactions with the FIB beam. Nevertheless, we know that the block face is constantly being ablated by the ion beam with removal of all C atoms as the implanted Ga layer moves slowly though the block while volume data are being acquired. To help explain the HAADF line profile, we include two dashed lines in Fig. 4b. The top dashed line corresponds to no loss of C and O atoms, i.e., the elemental composition of the Epon-Araldite remains intact with addition of Ga from the ion beam; and the lower dashed line corresponds to 50% loss of C and complete loss of O through ablation of atoms by interaction with the Ga^+^ ions. We can then obtain estimates of the Ga content of the implantation surface layer based on atomic cross section models for elastic scattering by Salvat et al.^20^ and Jablonski et al.^21^ computed based on the acquisition parameters for our HAADF STEM (Supplemental Table 1), in addition to our analysis (Supplemental Note 1, and Supplemental Fig. 1). If there is no loss of carbon from the region of high Ga implantation, the atomic ratio of Ga:C is 0.38:1, which corresponds to an atomic composition of 28 at. % Ga; 72 at. % C. However, if 50% of the C and all the O atoms are lost, the Ga:C ratio is 0.86:1, which corresponds to an atomic composition of 46 at. % Ga; 54 at. % C.

**Figure 5:**
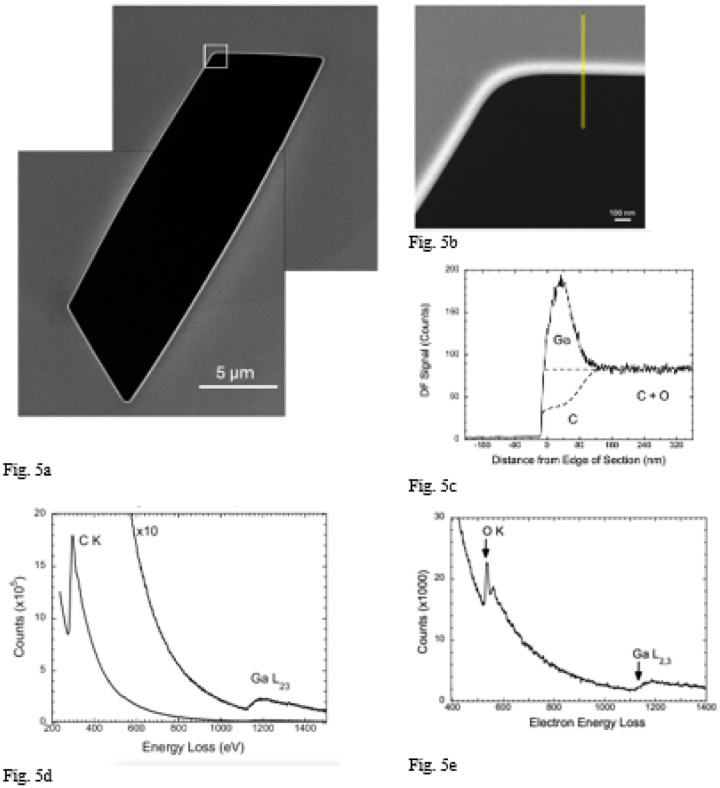
STEM-HAADF image of FIB-milled Epon section cut to thickness of ~200-nm with an ultramicrotome together with line profile, and EELS analysis of the intensity at the milled edge. **(a,b,c,d,e)** As in Figure 4 for the sectioned block face, the HAADF image shows a higher intensity at the milled edge of the Epon section in a low magnification image of the entire milled region which has a length of ~16 μm (**a**). A higher magnification image of the square region of interest in (**a**) reveals a broader intensity at the edge of the milled section (**b**), and a line profile showing a peak of width ~80 nm (**c**). The line profile is wider but less intense, which can be attributed to the lack of a Pt pad so that the size of the FIB-beam is no longer constrained. The larger width of the bright line facilitated EELS analysis since beam damage and contamination of the specimen could be reduced; the energy loss spectrum shows the presence of the C K edge and the Ga L_2,3_ edge (**d**). Quantitative analysis was calibrated by using Ga_2_O_3_ particles as a reference standard, and by computing the ratio of core-edge intensities for the Ga L_2,3_ edge and the C K edge, as well as the ratio of scattering cross sections for the C K edge and the O K edge as explained in the text (**e**).

We also obtained HAADF images from sectioned Epon-Araldite blocks that were deposited on EM grids and mounted in the specimen stage of the Zeiss Crossbeam 550 FIB-SEM. Regions corresponding to trenches in the FIB-milled blocks were milled in the sections as shown in Fig. 5a, where a bright HAADF signal is observed at the milled edges of the 2-D trench. A higher magnification view of the boxed region in Fig. 5a is shown in Fig. 5b, where the location of a line profile across the milled edge is indicated; the profile is plotted in Fig. 5c. Again, there is a strong peak in the HAADF signal at the edge. However, the peak in the line profile is much broader (~80 nm) than the peak in the section through the FIB-milled block in Fig. 4b (~20 nm), and the jump ratio of the Ga peak relative to the signal from the Epon-Araldite is about a factor of two lower (ℛ~2.26:1). We attribute the broader peak to the absence of a protective pad in the milling of the sections.

### 3. STEM-EELS analysis of FIB-milled sections of Epon-Araldite

Although the concentrations of Ga at the milled edges of ~200 nm thick Epon-Araldite sections were lower than in the sectioned FIB-SEM block faces, as reflected by the ratio of intensities of HAADF signal at the milled edge of the section relative to the signal in the Epon-Araldite far from the milled edge, we were able to use these samples to perform EELS analyses of the implantation layer. We were not able to obtain useful EELS data from the sectioned FIB-SEM block faces, due to the narrower width of the Ga-implanted layer, and radiation damage of the specimen at fluences required to obtain Ga L_2,3_ edge spectra at energy losses above 1,140 eV.

Electron energy loss spectra were acquired at a beam energy of 300 keV from the milled Epon-Araldite sections using a Tecnai TF30 TEM, which was also equipped with a Gatan Quantum Dual EELS imaging filter controlled by the Gatan Digital Micrograph software. EELS data were collected in STEM-EELS spectrum-imaging mode over an energy loss range from 200 eV to 1600 eV (Fig. 5d). Elemental images containing about 1,000 pixels were acquired with a pixel size of 2 nm, probe current of ~1 nA, and acquisition time of ~0.1 s/pixel. Spectra integrated over areas of several hundred nm^2^ were quantified using reference spectra from Ga_2_O_3_ nanoparticles deposited on lacy carbon support films (Fig. 5e), and by measuring the core-edge signal for the C K edge, Ga L_2,3_ edge, and O K edge (Figs. 5d, 5e; Supplemental Note 2). The energy window used for all inner shell edges was 100 eV and collection semi-angle was 10 mrad. The ratio of core edge cross section for the O K edge to the cross section for the C K edge was obtained from the EELS analysis module of the Gatan Digital Micrograph program (Supplemental Fig. 2). Averaging EELS data from 10 regions of the milled edge of the Epon-Araldite section, we find an atomic Ga:C ratio of 0.117±0.012:1, corresponding to 10.5 at. % Ga, whereas analysis of the HAADF image indicates that the atomic Ga:C ratio is 0.185:1, which corresponds to 15.6 at. % Ga, assuming that no carbon is lost from the specimen. If 50 % of the C atoms are lost from the specimen, the atomic Ga:C ratio would be 0.46:1, which corresponds to 31.5 at. % Ga. In view of the high fluence of > 10^8^ e/nm^2^ required to perform the STEM-EELS measurements it is possible that some of the Ga atoms are displaced resulting in a lower Ga concentration than is evident in the HAADF images acquired at a 5 orders of magnitude lower fluence of ~10^3^ e^−^/nm^2^.

Although the STEM-EELS data do not allow us to quantify accurately the Ga content of the FIB milled edge of the Epon-Araldite sections, the measurements are useful in that they confirm that a substantial amount Ga is embedded in the edge region of the milled section, as evidenced by the detection of the Ga L_2,3_ edge at 1,140 eV.

### 4. Monte Carlo simulations of z-resolution in stained structures embedded in Epon-Araldite with and without Ga^+^ ion implantation

**Figure 6:**
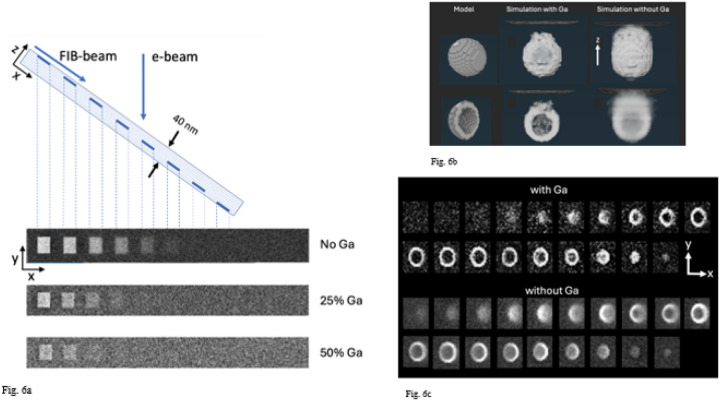
Monte Carlo simulations of backscattered electron images from regions of 3% atomic concentration of heavy metal (Pb) stain in an Epon block using the software MC XRAY1.7. (Gauvin & Michaud^22^). **(a,b,c)** The simulations were performed for an electron beam energy of 1.5 keV, and 3,000 trajectories per pixel. The contrast from the regions of stain were computed for Epon resin without Ga-ion implantation, with 25 atomic % Ga implantation, and with 50 atomic % Ga implantation. The stain was located at depths of 0–5 nm, 5–10 nm, 10–15 nm, 15–20 nm, and 20–25 nm below the surface (**a**). With no Ga implantation, five regions are visible indicating a z-resolution of 20–25 nm; with 25% Ga implantation, three regions are visible indicating a z-resolution of 10–15 nm; and with 50% Ga implantation, two regions are visible, indicating a z-resolution of 5–10 nm. A Monte Carlo simulation of the contrast of a single synaptic vesicle of diameter 40 nm with a membrane of thickness 5 nm (model) was computed for a Ga concentration of 50 atomic % and without Ga (**b**). The Monte Carlo simulation with Ga predicts considerably greater spatial resolution than the simulation without Ga implantation. The simulated images with and without Ga implantation are shown in the panels in (**c**). Without Ga implantation the contrast is reduced substantially, and the stained vesicle becomes visible when it is situated below the surface, demonstrating loss of z-resolution. The voxel size in simulation was 3 nm × 3 nm × 3 nm.

Having established from our HAADF-STEM measurements that the top 20 nm layer of FIB-SEM Epon-Araldite blocks contains high levels of gallium implantation, between ~25 at. % and ~50 at. % Ga, with the remaining atoms being mainly C, we ran Monte Carlo simulations to investigate the effect of the Ga on the quality of images obtained from stained biological structures using the software MC XRAY1.7. of Gauvin & Michaud^22^, based on earlier work by Hovington et al.^23^, Drouin et al.^24^, and Hennig & Denk^25^. We first considered a model structure consisting of Epon-Araldite in which 5-nm-thick square blocks of Pb at an atomic concentration of 3% were placed at different depths at thickness intervals of 5 nm. This concentration of approximately 3 atomic % of heavy elements associated with biological structures in specimens prepared for vEM^26^ had been previously estimated by He et al.^9^. The simulations were run (1) without any Ga implantation, (2) with 25 atomic % of Ga implantation, and (3) with 50 atomic % of Ga implantation. The stain was located at depths of 0–5 nm, 5–10 nm, 10–15 nm, 15–20 nm, and 20–25 nm below the surface. For each pixel in the simulated images 3,000 electron trajectories were computed. With no Ga implantation, five regions are visible indicating a z-resolution of 20–25 nm; with 25% Ga implantation, three regions are visible indicating a z-resolution of 10–15 nm; and with 50% Ga implantation, only two regions are visible, indicating a z-resolution of 5–10 nm (Fig. 6a). In these simulations it was assumed that carbon atoms are replaced by gallium atoms. The results explain the previously puzzling finding that the z-resolution in FIB-SEM in the direction perpendicular to the block face is much higher than expected for primary electrons of energy 1.5 keV, which we use to acquire volume data in our laboratory. With 50 at. % gallium, the simulations show that the resolution is between 5 and 10 nm. Fig. 6a also reveals that although the Ga reduces the contrast, there is sufficient signal-to-noise to detect the stained squares in the top 10 nm. Importantly, the simulation in the absence of Ga does not correspond to a real specimen because the specimen (e.g., in the SBF-SEM) would collapse under the electron fluence > 10^3^ nm^−2^. In fact, we have shown samples analyzed by FIB-SEM can tolerate fluences > 10^4^ nm^−2^. This resistance of FIB-SEM specimens to damage is consistent with the findings of Samira et al.^27^, who showed that Ga^+^ FIB beam interactions with epoxy resin could be used to microfabricate cantilevers for use in atomic force microscopy (AFM) and that these structures had a Young’s modulus of between 30 and 100 GPa, which could explain the exceptional stability of specimens that are imaged in the FIB-SEM.

We have found that FIB-SEM data sets allow us to visualize the 3D structure of individual synaptic vesicles in neuronal terminals (Supplemental Movie 1). Synaptic vesicles are spherical organelles of diameter ~40 nm bounded by phospholipid bilayer membrane containing various membrane proteins, inside which are stored neurotransmitter molecules whose controlled release triggers neuronal activity. We have modeled the vesicle membrane as a shell of thickness 5 nm containing heavy atoms of stain at a concentration of 3 atomic %, and we have performed Monte Carlo simulation of FIB-SEM image stacks with and without 50 atomic % Ga implantation, using a voxel size of 3 nm in x, y, and z. The model that includes Ga implantation predicts considerably greater spatial resolution than the simulation without Ga^+^ implantation (Fig. 6c). Without Ga^+^ implantation the contrast is reduced substantially, and the stained vesicle becomes visible when it is situated below the surface. This result demonstrates that Ga^+^ ion implantation explains the improved z-resolution in FIB-SEM relative to the predicted z-resolution if there were a low level of Ga^+^ ion implantation.

## Discussion

By analyzing the composition at the surface layer of the block face in stained biological samples embedded in epoxy resin, we can now understand how Ga^+^ FIB-SEM provides 3D ultrastructure of cells and tissues with high resolution perpendicular to the block face. In the FIB-SEM we can use electron fluences as high as 10^4^ e^−^/nm^2^, whereas the same specimen can only be imaged in SBF-SEM at a fluence of ~20 e^−^/nm^2^, otherwise the block collapses under electron irradiation faster than the diamond knife can section. The gallium implantation can reach levels of 40 or 50 atomic % within a 20-nm wide layer of the surface, which is consistent with the microfabrication of AFM cantilevers reported by Samira et al.^27^, and the formation of a glassy phase with a high Young’s modulus. Hard skins on polymer samples formed by interaction with Ga^+^ FIBs were first observed by Moon et al.^28^. It seems that a similar interaction occurs over the entire block face in FIB-SEM imaging of epoxy resin-embedded biological specimens, thereby converting the stained biological structures into a strong inorganic material that can withstand beam damage, and preventing the 1.5 keV electron probe from penetrating to the subsurface layer, resulting in a z-resolution of 5–10 nm rather than an expected resolution of ~25 nm if a much lower level of gallium were implanted. The higher tolerated electron fluence of 10^4^ e^−^/nm^2^ enables features in the images to be visualized, even though the intrinsic contrast is decreased by the gallium implantation; this is achievable because the signal-to-noise ratio is sufficient to detect features having relatively weak contrast. In addition, the gallium implantation increases the electrical conductivity of the entire block face preventing the specimen from charge build-up, even when individual cells are dispersed and surrounded by pure epoxy resin, which offers no path to ground.

We know that a transient high Young’s modulus Ga-rich material can be produced at the block face^27^, which is completely ablated under continuing exposure to the Ga^+^ FIB. This leads us to conclude that the 20-nm wide surface layer persists long enough for collection of the ultrastructural images close to the surface. Our results suggest that continued exposure to the Ga^+^ ion beam lowers the carbon concentration to a critical value, below which the structure becomes unstable resulting in a phase transition with complete loss of material from the surface, i.e., ablation. Meanwhile, the radiation-resistant surface layer moves further into the block enabling collection of ultrastructural images from the deeper layers underlying the initial block face.

Our results further suggest that biological FIB-SEM using a plasma ion beam, e.g., He^+^, Ne^+^, Xe^+^ might not provide such a high z-resolution because of the lower average atomic number of the biological matrix in the absence of Ga^+^ ion implantation. Furthermore, the specimens are likely to be more susceptible to damage by the electron beam, similar to imaging in the SBF-SEM, even though artefacts due to the gallium implantation are avoided^29^.

Finally, one might speculate that, had not the Ga^+^ FIB-SEM already been invented as a lift-out preparation technique for inorganic materials characterization by transmission electron microscopy, the same tool might have been designed specifically for volume EM. The design criteria would include (1) reducing the range of distances below the surface of the epoxy resin-embedded specimen block from which low-energy (~1.5 keV) backscattered electrons are generated by implanting a high concentration of an element with intermediate atomic number; (2) avoiding electric charge build-up by implanting a metallic element; and (3) converting the radiation-sensitive epoxy resin containing the heavy atoms of stain (i.e., Os, Pb, and U) into a strong, rigid, and radiation resistant inorganic glassy phase. One might expect that the optimal atomic number of the implanted ion should be roughly the geometric average of the atomic number of the light organic elements contained in the epoxy resin plus embedded biological material (Z_org_) and the atomic number of stain (Z_stain_). This would ensure that the electron range is reduced by a substantial factor, while also ensuring that the atoms of stain are still visible when the implanted ion has a high concentration. We might then expect that:

$$Z_{\text{implanted}-\text{ion}}\approx \sqrt{Z_{\text{organic}}Z_{\text{stain}}}$$

Taking a mean value of *Z*_*organic*_ = 7 (ignoring H atoms), and *Z*_*stain*_ = 82 (the mean value for Os, Pb, and U atoms), we predict an optimal value for $Z_{\text{implanted}-\text{ion}}\approx \sqrt{7\times 82}\approx 24$, which is relatively close to *Z*_*Ga*_ = 31 This choice of atomic number for the implanted ion thus ensures that the electron range is reduced by a substantial factor, while also ensuring that the atoms of stain are still visible above the background of the implanted ions.

The fact that the Ga^+^ FIB-SEM works so well for vEM is serendipitous, since the original applications of Ga^+^ FIB-SEM involved imaging the ultrastructure of inorganic materials^30^, as well as preparing lamellae for high-resolution TEM imaging based on the lift-out technique^31^. Applications were directed to metals, semiconductors, and ceramics, rather than to biological specimens. But, unlike the case of inorganic materials, where Ga^+^ ion implantation is considered as an artifact near the ablated surfaces, we have demonstrated that Ga^+^ ion implantation in vEM of biological specimens serves an essential role in modifying the specimen to improve the z-resolution, to eliminate charge build-up, and to greatly reduce radiation sensitivity. In fact, in its current form, FIB-SEM of embedded eukaryotic cells and tissues would not work without the Ga^+^ ion implantation.

## Methods

### Sample Preparation.

Dissociated rat hippocampal neurons was plated on glia cells pre-plated on acid cleaned and poly-lysine coated glass substrate and in a custom growth media in 35 C and 5% CO_2_ for 21 days before being fixed in 4% glutaraldehyde for 1 hr at RT. Samples were further fixed using 0.1 M cacodylate buffer containing 2.5% glutaraldehyde and 2 mM CaCl_2_ for 1 h in ice, and they were then washed three times with cold 0.1 M sodium cacodylate buffer containing 2 mM CaCl_2_ and spun at 600 × g for 5 min. Samples were then fixed in 3% potassium ferrocyanide in 0.3 M cacodylate buffer with 4 mM CaCl_2_ combined with an equal volume of 4% osmium tetroxide for 1 h in ice. After washing five times with H_2_O samples were then placed in a 0.22 μm-Millipore-filtered 1% thiocarbohydrazide (TCH) solution in ddH_2_O for 20 min following five washes with double-distilled water (ddH_2_O) at RT each for 3 min. Samples were fixed in 2% osmium tetroxide in ddH_2_O for 30 min at RT following five washes with ddH_2_O at RT each for 3 min and then placed in 1% uranyl acetate (aqueous) for overnight at 4°C. The next day, samples were washed five times with ddH_2_O at RT each for 3 min and processed for *en bloc* Walton’s lead aspartate staining at 60°C for 30 min following five washes with ddH_2_O at RT each for 3 min. Samples were dehydrated before resin embedding. Blocks of Epon Embed-812 /Araldite-502 resin (Electron Microscopy Sciences, Hatfield, PA) having the same composition as blocks containing embedded biological samples in our laboratory, were prepared by mixing Embed-812 (25 mL), Araldite 502 (15 mL), DDSA (55 mL), BDMA (2.5 mL). The elemental composition of the embedding resin is provided in Supplemental Table 2.

### FIB-SEM.

Specimens were imaged in a Crossbeam 550 focused ion beam scanning electron microscope (FIB-SEM) (Carl Zeiss Microscopy LLC, Thornwood, NY). A trapezoidal-shaped trench was ablated by the Ga^+^ beam (set to a high current of ~30 nA) in front of the sample block with a narrower edge at the block face, i.e., at the location of the Pt pad, and a wider edge away from the block face (Figs. 1a and 1b) to allow access for the incoming 1.5 keV electron beam and the outgoing backscattered electrons from the block face. The block face was imaged with a 1.5 keV electron beam using an in-lens energy selective backscattered-electron (ESB detector) with the grid voltage set to 700 V to eliminate inelastically scattered electrons with multiple energy losses greater than 700 eV -- originating deeper in the specimen block -- from entering the detector. Images were acquired with concurrent 700 pA electron and 700 pA Ga^+^ FIB beams, and with a pixel acquisition time of 16 μs. In addition to performing FIB-SEM imaging on Epon-Araldite blocks, which provided block faces of width ~20 μm and height ~20 μm, we also placed thin ~200-nm ultramicrotomes slices of Epon-Araldite embedding material supported on EM grids into the stage of the FIB-SEM, tilted the grid to 54° to the horizontal and used the FIB to ablate the Epon-Araldite section using the same FIB current of 700 pA used to ablate the trench in the solid specimen block. Further details on the experimental set-up, important for understanding the rationale for the current study, are provided in the first section of the Results.

### SBF-SEM imaging.

Specimen blocks that had been prepared in situ in the FIB-SEM (above) were removed from the FIB-SEM and transferred to a Zeiss Sigma VP SEM (Carl Zeiss Microscopy LLC, Thornwood, NY), which was equipped with an in situ Gatan 3View (Gatan/Ametek, Warrendale, PA) serial block face ultramicrotome and an OnPoint backscattered electron detector, and a Carl Zeiss focal charge compensation (FCC) system to prevent specimen charging. The FIB-SEM block face was perpendicular to the surface of the specimen block (as indicated in Figs. 1a and 1b). The Pt pad was removed by cutting sections parallel to the surface of the specimen block. After the pad had been completely removed, the SBF-SEM block face contained the edge of the FIB-SEM block face. These images revealed an elevated backscattered electron signal at the edge.

### HAADF-STEM imaging.

Specimen blocks that had been prepared in situ in the FIB-SEM (above) were removed from the FIB-SEM and transferred to a Leica EM UC6 ultramicrotome (Leica Microsystems, Buffalo Grove, IL). After removal of the Pt. pad from the top of the specimen block, sections of thickness ~200 nm were cut through the trench and FIB-SEM block face. These sections were then deposited on square mesh copper EM grids. The sections were analyzed in an FEI/Thermo Fisher Tecnai TF30 TEM (Thermo Fisher Scientific, Hillsboro, OR) using dark-field STEM at a primary beam energy of 300 keV, and a Fischione Model 3000 high-angle annular dark field (HAADF) detector. The probe current was 160 pA with a pixel dwell time of 5 μs, and a nominal probe size of 2 nm, giving an estimated electron fluence of 1.2 × 10^3^ e^−^ nm^−2^. Dark-field images were also acquired from 200-nm thick Epon-Araldite films that had been placed in the FIB-SEM and subjected to ablation by the Ga^+^ FIB beam.

### EELS analysis.

EELS data were obtained from the 200-nm thick plastic sections that had been subjected to ablation by the FIB beam in the Zeiss Crossbeam 550 SEM. Electron energy loss spectra were acquired using the Gatan Digital Micrograph software in the STEM-EELS mode using the same Tecnai TF30 TEM used to collect the HAADF data. This instrument was also equipped with a Gatan Quantum Dual EELS imaging filter (Gatan/Ametek Inc., Warrendale, PA). The beam energy was 300 keV and using the Digital Micrograph program. The energy loss range was 200 eV–1600 eV, and the elemental maps were taken from small areas of the specimen. Elemental images containing about 20 by 20 pixels were acquired with a pixel size of 2 nm and with a probe current of ~1 nA and an acquisition time of ~0.1 s/pixel. The electron fluence was estimated to be ~1.5 × 10^8^ e^−^ nm^−2^. Electron energy loss spectra from the block face region were quantified using reference spectra from gallium oxide, which were collected from Ga_2_O_3_ nanoparticles (Thermo-Fisher Chemicals, Waltham, MA) deposited on lacy carbon support films, and by analyzing the C K edge and the Ga L_2,3_ edge for the block face region, and the O K edge and the Ga L_2,3_ edge for the Ga_2_O_3_ nanoparticles. The energy window used for all inner shell edges was 100 eV and collection semi-angle was 10 mrad. The ratio of core edge cross section for the O K edge to the cross section for the C K edge was obtained from the EELS analysis module of the Gatan Digital Micrograph program (Supplemental Fig. 2).

### Simulations of backscattered images.

The MC X-Ray 1.7 Monte Carlo simulation software was used to simulate electron backscattered images from stained biological structures embedded in Epon-Araldite blocks with and without gallium implantation. The software, which was downloaded from https://montecarlomodeling.mcgill.ca/download/download.html, is in the public domain^22^. MC X-Ray is an extension of the Monte Carlo programs CASINO and Win X-Ray since it computes the complete x-ray spectra from the simulation of electron scattering in solids of various types of geometries. In this study we only used the software to generate backscattered electron images. We simulated the backscattered signal from square regions containing 3 atomic % Pb, which we have shown to have the highest concentration of the three heavy elements, Os (Z=76), Pb (Z=82), and U (Z=92), present in specimens prepared by UC San Diego NCMIR protocol that we use in our lab (He et al.^9^), and the atomic number of Pb is close to the mean atomic number of the three elements of stain. In the simulations, the regions of stain are 5 nm in thickness and are placed at different depths below the surface at intervals of 5 nm. The composition of the embedding material is given in Supplemental Tables 2 and 3. The simulations were carried out with no gallium implantation, with 25 atomic % Ga, and with 50 atomic % Ga following the experimental estimates of the possible ranges of Ga to C, based on the HAADF STEM and EELS data. Monte Carlo simulations were also performed on a modeled presynaptic vesicle of outer diameter 40 nm, with a membrane of thickness 5 nm and a stain concentration of 3%, with a voxel size in x, y and z of 3 nm × 3 nm × 3 nm. Simulations were performed for no Ga implantation and for 50% Ga implantation.

## Supplementary Files

This is a list of supplementary files associated with this preprint. Click to download.

- Yangetal09292024SupplementalInformation.pdf
- VesicleAnimation.mpg

Supplemental information is appended to this paper.
